# Evaluation of the efficacy and safety of endovascular management for transplant renal artery stenosis

**DOI:** 10.6061/clinics/2017(12)09

**Published:** 2017-12

**Authors:** Leonardo G.M. Valle, Rafael N. Cavalcante, Joaquim M. Motta-Leal-Filho, Breno B. Affonso, Francisco L. Galastri, Marisa P. Doher, Nadia K. Guimarães-Souza, Ana K.N. Cavalcanti, Rodrigo G. Garcia, Álvaro Pacheco-Silva, Felipe Nasser

**Affiliations:** I Departamento de Radiologia Intervencionista, Hospital Israelita Albert Einstein, Sao Paulo, SP, BR; II Departamento de Nefrologia, Hospital Israelita Albert Einstein, Sao Paulo, SP, BR

**Keywords:** Transplant Renal Artery Stenosis, Endovascular Treatment, Stenting, Renal Transplantation

## Abstract

**OBJECTIVES:**

To evaluate the safety and efficacy of endovascular intervention with angioplasty and stent placement in patients with transplant renal artery stenosis.

**METHODS:**

All patients diagnosed with transplant renal artery stenosis and graft dysfunction or resistant systemic hypertension who underwent endovascular treatment with stenting from February 2011 to April 2016 were included in this study. The primary endpoint was clinical success, and the secondary endpoints were technical success, complication rate and stent patency.

**RESULTS:**

Twenty-four patients with transplant renal artery stenosis underwent endovascular treatment, and three of them required reinterventions, resulting in a total of 27 procedures. The clinical success rate was 100%. All graft dysfunction patients showed decreased serum creatinine levels and improved estimated glomerular filtration rates and creatinine levels. Patients with high blood pressure also showed improved control of systemic blood pressure and decreased use of antihypertensive drugs. The technical success rate of the procedure was 97%. Primary patency and assisted primary patency rates at one year were 90.5% and 100%, respectively. The mean follow-up time of patients was 794.04 days after angioplasty.

**CONCLUSION:**

Angioplasty with stent placement for the treatment of transplant renal artery stenosis is a safe and effective technique with good results in both the short and long term.

## INTRODUCTION

Renal transplantation is an important therapeutic option for patients with end-stage chronic kidney disease, and it is associated with increased rates of survival and a better quality of life in these patients 1.

However, certain complications following transplantation may affect the graft and patient survival. With advances in immunosuppressive drugs, graft loss due to rejection has decreased to rates of 20-30%. Additionally, other complications have also gained major importance 2. Transplant renal artery stenosis (TRAS) is the most common vascular complication that may occur after transplantation, affecting 1-23% of patients 3.

Vascular alterations in the renal artery of the transplanted kidney may be either asymptomatic and/or associated with cases of refractory hypertension during clinical treatment and/or graft dysfunction 6, and they are typically related to decreases in overall survival rates of patients who undergo renal transplantation 4-7.

The benefits of endovascular intervention for the correction of TRAS remain under debate, however, and few results are currently available in the literature. It is believed that this technique may aid in the control of high blood pressure and may improve renal function, contributing substantially to better graft and patient survival 3,8-10.

This study aimed to evaluate the safety and efficacy of endovascular intervention with angioplasty and stent placement in patients with TRAS.

## MATERIALS AND METHODS

### Study design

This was a retrospective study carried out in the Department of Interventional Radiology and Nephrology, and it was approved by the Ethics and Research Committee of the institution.

### Patients and eligibility

All patients diagnosed with TRAS who underwent endovascular treatment from February 2011 to April 2016 were included in this study.

The diagnosis of TRAS was confirmed via Doppler ultrasonography (peak systolic velocity >200-250 cm/s, resistance index >0.8, pulsatility index >1.5, stenosis/prestenotic velocity gradient >2:1, *tardus parvus* in systolic acceleration >0.1 seconds, or acceleration in renal hilum >100 cm/s).

All patients included in the study had either graft dysfunction or resistant systemic hypertension. Graft dysfunction was defined as delayed graft function in kidney transplants assessed by measuring the creatinine level and estimated glomerular filtration rate (eGFR) using the Chronic Kidney Disease Epidemiology Collaboration (CKD-EPI) equation, while resistant systemic hypertension was defined as blood pressure >140/90 mmHg, despite treatment with antihypertensive drugs.

### Endovascular procedure

Patients with stenosis diagnosed using Doppler ultrasonography underwent an angiographic study. The access route for the procedures was either the ipsilateral or contralateral common femoral artery. Pelvic and transplanted renal artery angiograms were performed for anatomical evaluation and lesion quantification.

Following angiographic confirmation of stenosis, systemic heparinization was performed with unfractionated heparin, followed by transposition of the lesion with a 0.014 guidewire. All cases were treated with stent implantation: in one case, a self-expanding stent was used, and in the other cases, expandable balloon stents, such as Vision^®^ Cobalt Chromium (Abbott Laboratories, Chicago, IL, USA), TAXUS™ Liberté™ (Boston Scientific Corporation, Natick, MA, USA), Express™ Vascular Stent (Boston Scientific Corporation), Dynamic Stent (BIOTRONIK, Berlin, Germany), and/or Direct-Stent^®^ (InSitu Technologies, St. Paul, MN, USA), were used. The materials were used without standardization at the discretion of the interventional physician. At the end of the procedure, hemostatic devices, such as the Perclose Proglide (Abbott) and/or the StarClose (Abbott) devices, were used at the puncture site.

### Clinical variables and outcomes

Information such as sex, age, risk factors, technical data related to transplantation (i.e., from both the donor and recipient), laboratory tests, diagnostic methods, anatomical pattern of stenosis, and technical details related to treatment (e.g., contrast volume, tomographic imaging, material used, treatment methods, postoperative assessment with arterial patency, blood pressure control, renal function, and survival) was collected. Renal function was determined using serum creatinine levels, and the eGFR was obtained in the preoperative and postoperative periods (i.e., 48 hours prior and 30, 60, and 90 days after) via the CKD-EPI equation.

Technical success was defined as revascularization with stent implantation without complications and/or with complete absence of or <30% residual stenosis. Clinical success was defined as improved renal function (both creatinine levels and eGFR) in cases of renal dysfunction or improved blood pressure control, with a reduction in the use of antihypertensive drugs, in patients with arterial hypertension secondary to TRAS.

### Statistical analysis

The quantitative characteristics evaluated in the patients are described using summary measures (i.e., mean, standard deviation, median and quartiles), and the qualitative variables are described using absolute and relative frequencies.

The creatinine levels were compared preoperatively using the lowest value at follow-up via the paired Wilcoxon test. Blood pressure controls, measured mainly by the total number of medications used, were also compared between the preoperative and follow-up periods using the paired Wilcoxon test. A Kaplan-Meier analysis was used to determine the graft survival curve and to evaluate patency rates and the probability of occurrence of events. A *p*-value ≤0.05 was considered significant.

## RESULTS

From February 2011 to April 2016, 29 patients who were diagnosed with TRAS using Doppler ultrasonography underwent angiography. Twenty-four of these cases underwent endovascular treatment, and three required reinterventions, resulting in a total of 27 procedures.

Five patients underwent only diagnostic angiography without intervention and, therefore, were excluded from the final analysis. In four of these cases, angiography showed only mild stenosis with no flow change, while the remaining patient demonstrated severe stenosis and thrombosis of segmental branches, with changes in the parenchymal phase of the angiographic study. No treatment approach was indicated for this patient.

### Patient profile

The profile of the study population and the data concerning the renal grafts, as well as the surgical procedures used, are described in Table 1 and Table 2.

Of the 24 patients who were diagnosed with TRAS and underwent endovascular treatment, 21 had graft dysfunction and 3 had resistant systemic hypertension. Three of these patients underwent re-stenosis due to graft dysfunction.

All arterial anastomoses were performed on the iliac arteries. In one case, anastomosis was performed on the internal iliac artery (4.5% of cases), while anastomosis was performed on the external iliac artery in the remaining cases.

Of the 24 grafts used in the study, six (25%) grafts were from living donors. Furthermore, only one graft used during a reintervention was from a living donor (33% for reintervention).

### Technical and clinical success

The technical success rate of the procedure was 97%. The mean pretreatment and post-treatment stenosis rates were 79.40% and 2.40%, respectively. No relationship between the postoperative period and the stenosis site was found (Table 3).

**Figure 1:**
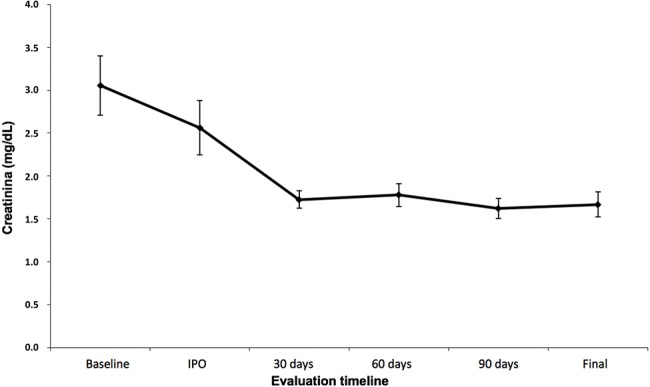
Preoperative and postoperative mean serum creatinine levels (in days).

**Figure 2:**
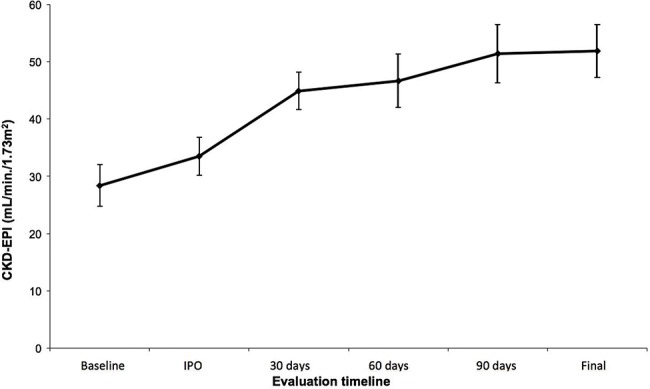
Preoperative and postoperative mean creatinine clearance rates (CKD-EPI) (in days).

The clinical success rate was 100%. All patients showed decreased serum creatinine levels (Figure 1) and improved eGFR and creatinine levels (Figure 2), with a statistically significant improvement noted following 30 days of treatment (*p*<0.001), which was further maintained after 60 and 90 days (Table 4). Patients with high blood pressure also showed improved control of systemic blood pressure and decreased use of antihypertensive drugs (*p*<0.008) (Table 5).

### Procedures and patency

The median diameter and length of the stents used were 5 and 12 mm, respectively (Table 6). Intraoperative tomographic imaging was performed in 37% of the cases, with no significant increase in contrast volume or radioscopy time noted (Table 7).

**Figure 3:**
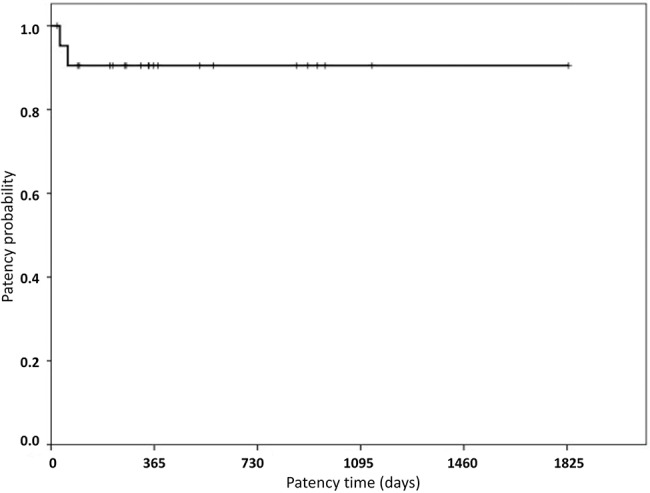
Patency probability following the procedure (in days).

The primary patency and assisted primary patency rates in one year were 90.5% and 100%, respectively. Both rates were maintained until the end of the study, considering the mean follow-up time of patients of 794.04 days after angioplasty (Figure 3). During the evaluation period, two patients died due to non-renal causes; their grafts were patent and functioned properly for 587 days and 1,023 days, respectively.

### Re-interventions

Of the three cases requiring re-intervention, one was due to in-stent re-stenosis after two months of treatment (Dynamic Stent 5×12, BIOTRONIK, Berlin, Germany). In this case, the patient’s renal dysfunction remained after stent placement. A new Doppler ultrasonography exam showed increased resistance in the renal artery. Angioplasty using an IntraStent^®^ balloon catheter (Covidien, Dublin, Ireland) was subsequently performed.

The second case of re-intervention was due to graft dysfunction. Three renal arteries were transplanted, with one branch initially treated with a stent (Vision^®^ Cobalt Chromium 3.0×15, Abbott Laboratories) and two branches treated with a pharmacological balloon catheter (FALCON 3×40). During the follow-up, five months after angioplasty, the patient’s serum creatinine levels were found to have increased, and a stent was placed in the two branches initially treated with a balloon (Vision^®^ Cobalt Chromium 2.0×28, Abbott Laboratories), with progression occurring favorably.

The third case underwent re-intervention initially due to graft dysfunction. The patient underwent endovascular treatment with stent placement (Dynamic Renal Stent, BIOTRONIK, Berlin, Germany) and, after an initial improvement of symptoms, exhibited further worsening of renal function. A second intervention was performed in which renal stent patency was observed, although with dissection of the left iliac artery, which was deemed to be a possible complication related to the first procedure. The patient underwent placement of a self-expanding stent (Everflex™, Covidien, Dublin, Ireland) in the iliac artery, and renal function improved.

### Complications

No immediate complications were observed after the procedure. A case of dissection of the common iliac artery with extension to the external iliac artery was diagnosed 30 days after the initial procedure but was resolved with a self-expanding stent.

## DISCUSSION

This retrospective study was carried out to evaluate the impact of endovascular treatment in patients diagnosed with TRAS, which was proven to be an effective and safe procedure.

The prevalence of TRAS observed in our study was 5.6%, similar to that found in other studies 3,5,6,11. Our analysis revealed renal dysfunction, associated with abnormalities in Doppler ultrasonography, to be a major clinical presentation and to be present in 90% of cases, a finding similar to that in other reviews 4,5.

A higher peak systolic velocity and velocity gradient between the transplanted renal artery and the iliac artery is a criterion of higher sensitivity in Doppler ultrasonography for TRAS 6. Although some authors cited the presence of a peak systolic pressure gradient >10% or stenosis >50% in angiography as an indication for intervention, no consensus has been established in the literature regarding stenosis ranges and/or standards for intervention 12. In this study, Doppler ultrasonography was used as a screening method in patients with a clinical presentation of TRAS.

The anastomosis site and onset may be related to different risk factors. The mean onset of TRAS in our study was 169 days, and the most common site of stenosis was the anastomosis, which is similar to findings described in other studies, and was present in more than half of the cases in this study 4,6,13. This stenosis may be associated with mechanical lesions of the blood vessels during organ recruitment or the surgical procedure and was found to present early after transplantation 14,15. The stenosis that occur later, sometimes several years after transplantation, usually reflect atherosclerotic disease and are also associated with rejection and/or cytomegalovirus infection 4,5,16,17. In our study, no relationship was found between the anastomosis site and time of onset. A review of the medical records showed that no vascular lesion was reported during organ recruitment or the surgical procedure, although other changes not described during the procedure, such as hyperflexion of blood vessels and endothelial injury during graft perfusion, are related to this type of stenosis.

The treatment of TRAS is crucial, as the presence of graft dysfunction and/or high blood pressure may alter the renal graft and compromise patient survival 10,18-20.

Concerning therapeutic options, clinical and medical care is limited to patients with TRAS, stable renal function, and non-significant lesions, while surgical intervention should be reserved as a salvage option in cases in which angioplasty is unsuccessful 9. Endovascular intervention, despite risks such as renal artery disease, re-stenosis, thromboembolism and complications related to the puncture site, may be considered a first-line therapy for TRAS 9,21,22.

Our results are similar to those in the literature, which describe a technical success rate ranging from 89 to 100% 4,16,21,23. In our study, the technical success rate was 97% and the clinical success rate was 100%.

Ngo et al. systematically reviewed 32 studies of TRAS and found a clinical success rate between 65.5 and 94% 24. Among the 28 studies that evaluated renal function, the mean creatinine level reduced 0.45 mg/dL after 30 days and 0.82 mg/dL after six months. Estimated GFR was evaluated in 11 studies, which showed an average gain of 8.6 mL/min/1.73 m^2^ three months after endovascular intervention. Our review showed that renal function significantly improved following intervention, with values higher than those found in these studies. The mean creatinine level decreased by 1.32 mg/dL and the eGFR increased by 16.53 mL/min/1.73 m^2^ after 30 days. These values were maintained after 90 days, and a short-term improvement in renal function was predictive in the long run. The mean follow-up of patients was 794 days, and all of them maintained stable renal function until the end of the follow-up period. The maintenance of satisfactory kidney function after transplantation is directly related to the graft and patient survival rates, and we expect that these factors may have an effect on our patients 18,19.

Among the patients undergoing treatment because of graft dysfunction, three underwent intervention due to delayed graft function (DGF), as they remained on dialysis even after renal transplantation. All of them left dialysis and maintained adequate renal function after correction of TRAS, which had an important effect on graft and patient survival rate, as DGF is associated with poor outcomes 19,25.

Despite the heterogeneous results found following endovascular treatment of TRAS with balloon catheters, studies with stent placement showed that high blood pressure was improved following this treatment, with a reduction of antihypertensive drugs, which was also found in our study 24,26. Adequate blood pressure control is very important, as high blood pressure can result in a decreased graft survival rate and left ventricular hypertrophy, the latter of which is an independent risk factor for heart failure and death in both the general population and renal transplant recipients 20,27.

During follow-up, patency levels ranging from 63 to 82% were described one year after endovascular treatment, with a re-stenosis rate ranging from 10 to 36% 28. In our study, a primary patency of 90.5% was reached after three months, and assisted primary patency of 100% was achieved after two years following angioplasty with a balloon-expandable stent.

In the literature, early complications are noted, including bleeding, pseudoaneurysm formation, thrombosis, arterial dissection, hematoma, and intimal flap, in approximately 10% of cases 6,29. In our experience, no cases of early complications were reported; however, it was observed that late complications occurred in 4% of patients, represented by one cases of iliac artery dissection.

No randomized trials comparing multiple treatment techniques for TRAS were found in the literature. This study is limited mainly because it is not a multicenter retrospective study, although our population was larger than that in many studies found in the literature.

Based on our experience, we conclude that angioplasty with stent placement for the treatment of TRAS is a safe and effective technique for improving eGFR, serum creatinine levels, high blood pressure control and graft survival rate, with excellent results in both the short and long term.

## AUTHOR CONTRIBUTIONS

Valle LG conceived and designed the study and was responsible for the analysis and interpretation, data collection, manuscript writing, critical revision of the manuscript, statistical analysis, final approval of the manuscript and overall responsibility. Cavalcante RN and Motta-Leal-Filho JM were responsible for the analysis and interpretation, manuscript writing, critical revision of the manuscript, statistical analysis, final approval of the manuscript and overall responsibility. Affonso BB conceived and designed the study and was responsible for the critical revision of the manuscript, final approval of the manuscript and overall responsibility. Galastri FL conceived and designed the study and was responsible for the analysis and interpretation, manuscript writing, final approval of the manuscript and overall responsibility. Doher MP was responsible for the analysis and interpretation, data collection, manuscript writing, critical revision of the manuscript, statistical analysis, final approval of the manuscript and overall responsibility. Guimarães-Souza NK was responsible for the analysis and interpretation, critical revision of the manuscript, final approval of the manuscript and overall responsibility. Cavalcanti AK was responsible for the data collection, manuscript writing, final approval of the manuscript and overall responsibility. Garcia RG conceived and designed the study and was responsible for the critical revision of the manuscript, final approval of the manuscript and overall responsibility. Pacheco-Silva A was responsible for the critical revision of the manuscript, final approval of the manuscript and overall responsibility. Nasser F Conceived and designed the study and was responsible for the critical revision of the manuscript, final approval of the manuscript and overall responsibility.

## Figures and Tables

**Figure 4:**
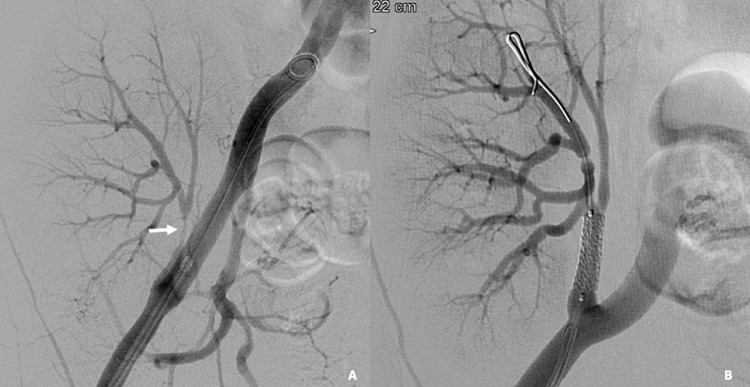
A 51-year-old female patient with acute graft dysfunction. (A) Angiographic study showing severe renal artery stenosis (arrow) in the segment distal to the anastomosis. (B) Control angiography after stenting, showing adequate positioning of an Express™ Vascular Stent 6×18 (Boston Scientific Corporation, Natick, MA, USA) with successful lesion treatment.

**Table 1:** Demographic Profile of Patients.

Variable	N	%
**Sex**		
Male	15	68.2
Female	7	31.8
**Hypertension**		
No	2	9.1
Yes	20	90.9
**DM**		
No	16	72.7
Yes	6	27.3
**CVD**		
No	18	81.8
Yes	4	18.2
Smoking*		
No	17	81.0
Yes	4	19.0
**Dyslipidemia**		
No	13	59.1
Yes	9	40.9
**Death**		
No	20	90.9
Yes	2	9.1
**Total**	**22**	**100**

* Information was not available for all patients.

DM: Diabetes mellitus.

CVD: Cardiovascular disease.

**Table 2:** Data Analysis Associated with the Renal Graft and Surgical Procedure.

Variable	Mean	SD	Median	Minimum	Maximum	N
Age	49.9	14.3	51.5	20.0	75.0	22
Days until TRAS	169.1	165.8	132.5	13.0	706.0	22
Donor age	42.6	13.9	43.5	19.0	69.0	22
Graft survival rate	689.5	462.9	575.5	115	2069	22
Radioscopy time (min)	20.4	11.8	16.1	7.2	56	18
Contrast volume (ml)	185.1	52.0	180.0	100	300	17

**Table 3:** Evaluation of the Stenosis Site.

Stenosis site	Days until TRAS						
	Mean	SD	Median	Minimum	Maximum	N	*p*
Anastomosis	190.23	212.82	135	13	706	13	0.794
Distal	138.56	49.81	130	86	246	9	
**Total**	**169.09**	**165.84**	**132.5**	**13**	**706**	**22**	

Mann-Whitney *U* test.

**Table 4:** Variation in Serum Creatinine Levels and Creatinine Clearance Rates (CKD-EPI) throughout the Study.

Variable	Time	Mean	SD	Median	Minimum	Maximum	N	*p*
Creatinine	Preoperative period	3.05	1.61	2.76	1.34	6.88	22	**<0.001**
	Immediate postoperative period	2.56	1.49	2.01	1.27	7.59	22	
	30 days	1.73	0.47	1.78	0.89	3.03	21	
	60 days	1.78	0.59	1.68	0.67	2.94	20	
	90 days	1.62	0.53	1.68	0.65	3.03	21	
	End of follow-up	1.67	0.68	1.68	0.80	3.49	22	
CKD-EPI	Preoperative period	28.39	17.18	23.26	7.48	68.15	22	**<0.001**
	Immediate postoperative period	33.49	15.64	31.84	6.93	63.00	22	
	30 days	44.92	15.06	40.85	21.47	76.57	21	
	60 days	46.71	20.78	42.41	23.62	103.90	20	
	90 days	51.41	23.38	41.11	21.47	107.78	21	
	End of follow-up	51.91	21.71	45.50	18.00	100.00	22	

Bonferroni correction.

**Table 5:** Preoperative and Postoperative Quantitative Analysis of Antihypertensive Drugs used by Patients.

Variable	Mean	SD	Median	Minimum	Maximum	N	*p*
Preoperative antihypertensive drugs	2.09	1.15	2	0	4	22	**0.008**
Postoperative antihypertensive drugs	1.59	1.05	1.5	0	4	22	

Wilcoxon signed-rank test.

**Table 6:** Evaluation of Patency Times and Stent Sizes.

Variable	Mean	SD	Median	Minimum	Maximum	N
Patency time (days)	488.91	450.09	345	21	1830	22
Mean diameter of stents	4.89	1.46	5	2	8	19
Mean length of stents	12.68	7.14	12	0	28	22

**Table 7:** Evaluation of Contrast Volume in Milliliters and Scoping Time Related to Cone-beam CT.

Variable	CBCT	Mean	SD	Median	Minimum	Maximum	N	*p*
**Contrast volume (ml)**	No	195.11	58.01	200	130	300	9	0.481
	Yes	173.75	45.336	175	100	250	8	
	**Total**	**185.06**	**51.986**	**180**	**100**	**300**	**17**	
**Radioscopy time (min)**	No	21.536	13.3556	17.765	10.5	56	10	0.762
	Yes	19.08	10.2365	16.06	7.2	34.5	8	
	**Total**	**20.444**	**11.7964**	**16.125**	**7.2**	**56**	**18**	

Mann-Whitney *U* test.
